# Abnormal Wave Reflections and Left Ventricular Hypertrophy Late After Coarctation of the Aorta Repair

**DOI:** 10.1161/HYPERTENSIONAHA.116.08763

**Published:** 2017-02-08

**Authors:** Michael A. Quail, Rebekah Short, Bejal Pandya, Jennifer A. Steeden, Abbas Khushnood, Andrew M. Taylor, Patrick Segers, Vivek Muthurangu

**Affiliations:** From the Centre for Cardiovascular Imaging, Institute of Cardiovascular Science, University College London and Great Ormond Street Hospital for Children, London, United Kingdom (M.A.Q., R.S., B.P., J.A.S., A.K., A.M.T., V.M.); Adult Congenital Heart Disease Department, St. Bartholomew’s Hospital, London, United Kingdom (B.P.); and IBiTech-bioMMeda, iMinds Medical IT, Ghent University, Gent, Belgium (P.S.).

**Keywords:** blood pressure, congenital heart disease, hemodynamics, hypertension, magnetic resonance imaging

## Abstract

Patients with repaired coarctation of the aorta are thought to have increased afterload due to abnormalities in vessel structure and function. We have developed a novel cardiovascular magnetic resonance protocol that allows assessment of central hemodynamics, including central aortic systolic blood pressure, resistance, total arterial compliance, pulse wave velocity, and wave reflections. The main study aims were to (1) characterize group differences in central aortic systolic blood pressure and peripheral systolic blood pressure, (2) comprehensively evaluate afterload (including wave reflections) in the 2 groups, and (3) identify possible biomarkers among covariates associated with elevated left ventricular mass (LVM). Fifty adult patients with repaired coarctation and 25 age- and sex-matched controls were recruited. Ascending aorta area and flow waveforms were obtained using a high temporal-resolution spiral phase-contrast cardiovascular magnetic resonance flow sequence. These data were used to derive central hemodynamics and to perform wave intensity analysis noninvasively. Covariates associated with LVM were assessed using multivariable linear regression analysis. There were no significant group differences (*P*≥0.1) in brachial systolic, mean, or diastolic BP. However central aortic systolic blood pressure was significantly higher in patients compared with controls (113 versus 107 mm Hg, *P*=0.002). Patients had reduced total arterial compliance, increased pulse wave velocity, and larger backward compression waves compared with controls. LVM index was significantly higher in patients than controls (72 versus 59 g/m^2^, *P*<0.0005). The magnitude of the backward compression waves was independently associated with variation in LVM (*P*=0.01). Using a novel, noninvasive hemodynamic assessment, we have shown abnormal conduit vessel function after coarctation of the aorta repair, including abnormal wave reflections that are associated with elevated LVM.

## Abstract 2

Supplemental Digital Content is available in the text.

Patients with repaired coarctation of the aorta (CoA) are thought to have increased afterload due to abnormalities in vessel structure and function. However, blood pressure, which is the simplest measure of afterload, is not universally elevated in these patients. This may simply be because of inaccuracies introduced when measuring blood pressure brachially rather than centrally. It is also possible that blood pressure itself is a limited measure of vascular and cardiac load after CoA repair. In particular, it may not adequately characterize arterial wave reflections that may be more prevalent in this group.

Characterization of central aortic hemodynamics and wave reflections is difficult, requiring simultaneous measurement of aortic pressure and flow. We have recently demonstrated the ability to assess central aortic systolic blood pressure (c-SBP) using a combination of phase-contrast magnetic resonance and oscillometric brachial artery blood pressure.^1^ We have also shown that it is possible to use the same high temporal-resolution phase-contrast magnetic resonance data to perform noninvasive wave intensity analysis (WIA).^2,3^ This allows the assessment of wave reflections, which has not previously been done in the CoA population.

Cardiac MR (CMR) also provides accurate assessment of left ventricular (LV) hypertrophy, which is known to be a risk factor for adverse cardiac events. Thus, using CMR, it is possible to accurately assess all aspects of conduit vessel function and determine their relationship to end-organ effects.

In this study, we recruited 50 patients with repaired CoA (without recoarctation) and 25 age- and sex-matched healthy controls. The aims of the study were to use high temporal-resolution CMR imaging to (1) characterize differences in c-SBP and peripheral systolic blood pressure (p-SBP) between patients and controls, (2) comprehensively evaluate afterload (including wave reflections) in the 2 groups, (3) identify possible biomarkers among covariates associated with elevated LV mass (LVM), and (4) substantiate in vivo observations with 1-dimensional (1D) computer simulations of wave travel in repaired coarctation.

## Materials and Methods

### Subjects

Fifty patients with CoA repaired in childhood and 25 healthy controls were recruited. Control subjects were volunteers from the same geographic area (Greater London) and were matched by age (within 2 years) and sex. Exclusion criteria were (1) irregular heart rates, (2) contraindications to CMR such as MR-incompatible implants, (3) pregnancy, (4) aortic stenosis, (5) coarctation associated with major or unrepaired congenital heart disease (exception nonstenotic bicuspid aortic valve or repaired ventricular/atrial septal defects), (6) coarctation stents, or (7) echocardiographic or CMR evidence of recoarctation (diastolic flow continuation in descending aorta or coarctation index [CI] <0.7). Patients receiving antihypertensive medications were included if this was reported as stable, chronic therapy. The study was performed with local research ethics committee approval and written informed consent was obtained.

### CMR Protocol

All imaging was performed on a 1.5-T MR scanner (Avanto, Siemens Medical Solutions, Erlangen, Germany) using 2 spine coils and 1 body-matrix coil. A vector electrocardiographic system was used for cardiac gating. The flow-imaging plane was planned using orthogonal long axis cine images of the ascending aorta and was placed just above the sinotubular junction (Figure S1, Line A). These data were used for the derivation of all subsequent hemodynamic indices. The sequence was a prospectively triggered, spiral, velocity-encoded spoiled gradient echo acquisition, accelerated with SENSE (TE/TR: 1.9/4.8 ms, FOV: 400×400×6 mm, matrix: 192×192, spiral interleaves: 60, SENSE factor: 4, VENC: 180 cm/s).^4^ The temporal resolution was 9.6 ms, the spatial resolution was 2.1×2.1 mm, and the breath-hold was ≈11 s (16 R–R intervals).

Aortic arch morphology was assessed in patients using gadolinium-enhanced MR angiography with a coronal 3D fast-field-echo sequence^5^ or a balanced, steady-state free precession sequence.^6^ A radial *k–t* SENSE sequence was used to calculate LV volumes and LVM as previously described (online-only Data Supplement).^7^

### Blood Pressure Measurement

Brachial SBP (p-SBP), diastolic BP (DBP), and mean BP were measured by automated oscillometric sphygmomanometry during flow imaging (Datex Ohmeda) on the patient’s right arm. Small-adult, adult, and large-adult cuff sizes were chosen according to subject arm circumference. Volunteers lay supine in the CMR scanner with the arm at the level of the heart, and there was a period of acclimatization (at least 15 minutes) before measurements were taken.

### Image Processing

All images were processed using an in-house plug-in for the open source DICOM software OsiriX (OsiriX Foundation, Geneva, Switzerland).^8^ Segmentation of the ascending aorta was performed on the modulus image using a previously validated semiautomatic registration-based algorithm (online-only Data Supplement).^9^

Aortic arch anatomy was evaluated by measurement of aortic diameter in the transverse aortic arch between innominate and left common carotid artery, distal aortic arch (repair site), and the descending aorta at the level of the diaphragm (Figure S1). Two metrics were derived from these measurements: (1) transverse arch index (TI), quantifying the degree of transverse arch hypoplasia: transverse arch diameter divided by descending aorta diameter. (2) CI, quantifying the degree of recoarctation: aortic isthmus (repair site) diameter divided by descending aorta diameter. Aortic arches were also characterized as “gothic” if the arch had an acutely angulated triangular conformation.^10^

### Derivation of c-SBP Using Area-Distension Waveforms

The aortic area waveforms were calibrated using a previously validated exponential pressure–area model and described below.^1^ The equation of an exponential pressure–area relationship is:^11,12^


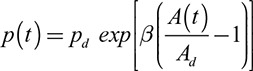


where *p(t*) is the synthesized pressure curve, *p*_*d*_ is the brachial DBP, *A*_*d*_ is the diastolic area, systolic *A(t*) is the area curve, and β is the scaling factor, initially set as follows:


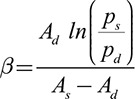


where *A*_*s*_ and *A*_*d*_ are the systolic and diastolic aortic areas, respectively. As c-SBP is, in general, lower than p-SBP, this initial starting β is the theoretical maximum.

Calibration of this model consisted of iteratively reducing the scaling factor β to minimize the difference between the measured brachial mean BP and the mean of the synthesized pressure curve. This calibration scheme was based on the validated assumption that the difference between DBP and mean pressure is constant in the large arteries.^13,14^ The estimated c-SBP was the peak of the optimized synthesized pressure curve.

### Wave Speed and Characteristic Impedance

Because of the possible early reflection site related to the repaired coarctation, conventional single cut methods of calculating wave speed (such as the Q/A method) are unreliable.^15,16^ Therefore, the Bramwell–Hill equation^17,18^ was used to obtain local pulse wave velocity, *c*, in the ascending aorta, using the aortic area waveform and c-SBP:


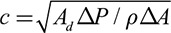


where *A*_*d*_ is the diastolic cross-section area, Δ*A* is *A*_*s*_−*A*_*d*_, and Δ*P* is the central pulse pressure (c-SBP–DBP). This method has been shown in computer simulations to be robust in the presence of early wave reflections.^15^ Characteristic impedance, Z_c_, was calculated by:


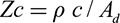


where *ρ* is assumed to be 1060 kg/m^3^.

### Wave Intensity Analysis

In WIA, waves are regarded as a summation of incremental wave fronts; it is, therefore, possible to separate the *Q* and *A* curves into the respective forward and backward components by expressing the relationship between wave speed and changes in flow and cross-sectional area, as previously described (Full methodology, online-only Data Supplement).^2^ Using this system, 4 different waves may be characterized: forward compression waves, forward expansion waves, backward compression waves (BCW), and backward expansion waves.

The type of wave and their magnitude (area under the wave) were determined by the analysis of the net and separated WIA plots in Matlab. The areas under the separated waveforms were calculated by numeric integration. Area waveforms were also separated into forward and backward components, by integration of dA_+_ and dA_−_ plots. Using these data, we calculated the reflection magnitude as: Area_backward_/Area_forward_.

### Arterial Resistance and Total Arterial Compliance

Arterial resistance (Woods Units) was calculated by dividing mean BP (mm Hg) by cardiac output (L/min). Total arterial compliance (TAC, mL mm Hg^−^^1^) was calculated in Matlab using a 2-element windkessel model as previously described,^19^ using single cardiac cycle phase-contrast flow curves and both central (TAC_central_) and brachial (TAC_brachial_) pulse pressure (SBP–DBP). Briefly, the aortic flow curve was inputted into the model with measured arterial resistance, and TAC was tuned so that pulse pressure generated by the model equaled measured pulse pressure. Resistance was indexed to body size by multiplication with BSA and TAC by dividing by BSA.

### One-Dimensional Computer Simulations

A validated 1D model of the systemic vascular tree^20^ was used to explore differences in wave reflection between controls and patients after coarctation repair. The model solves the 1D Navier–Stokes equations and provides pressure and flow waveforms along the arterial tree. The model was run using a time-varying elastance model for the heart on its upstream boundary, such that simulated waves originate from the interaction of the pumping heart in the arterial tree. Apart from the baseline condition using default parameters for the model (default lengths, inlet and outlet diameters and arterial distensibility), 2 additional scenarios were simulated. First, the diameter of a segment in the descending aorta was reduced by 25% to mimic a mild residual aortic coarctation, while maintaining the normal distensibility. Second, the stiffness of the narrowed section was increased by 5 orders of magnitude to mimic the effect of a stiffened coarctation repair zone.

### Statistics

STATA 13.1 and Graphpad Prism 5f were used for statistical analysis and figures. Data were examined for normality and where appropriate, non-normally distributed variables were log transformed to ensure normal distribution before analysis. Descriptive statistics are expressed as mean (±95% confidence interval) when normally distributed, and geometric mean (±95% confidence interval of geometric mean) when non-normally distributed, unless specified. Proportions are expressed as percentages.

The independent samples *t* test was used to compare differences in parametric data between coarctation patients and controls; Welch correction was used for unequal variances. Proportions test was used to compare proportions among groups. The level of α considered for statistical significance was 0.05.

Pearson correlation coefficient was used to analyze simple linear relationships between measures of arch morphology and hemodynamics. Multivariable linear regression analysis was also used to determine covariates independently associated LVM. The model was adjusted for: BSA, sex, age to ascertain independent associations. We also adjusted for case/control to control for unmodeled covariates.

## Results

### Study Population Characteristics

Demographics and conventional clinical measures in the patients and controls are shown in Table 1. There were no significant differences in age, sex, or ethnicity between patients and controls. Control subjects were taller on average than patients (*P*=0.03); however, there was no difference in BMI or BSA between groups (Table 1).

**Table 1.**
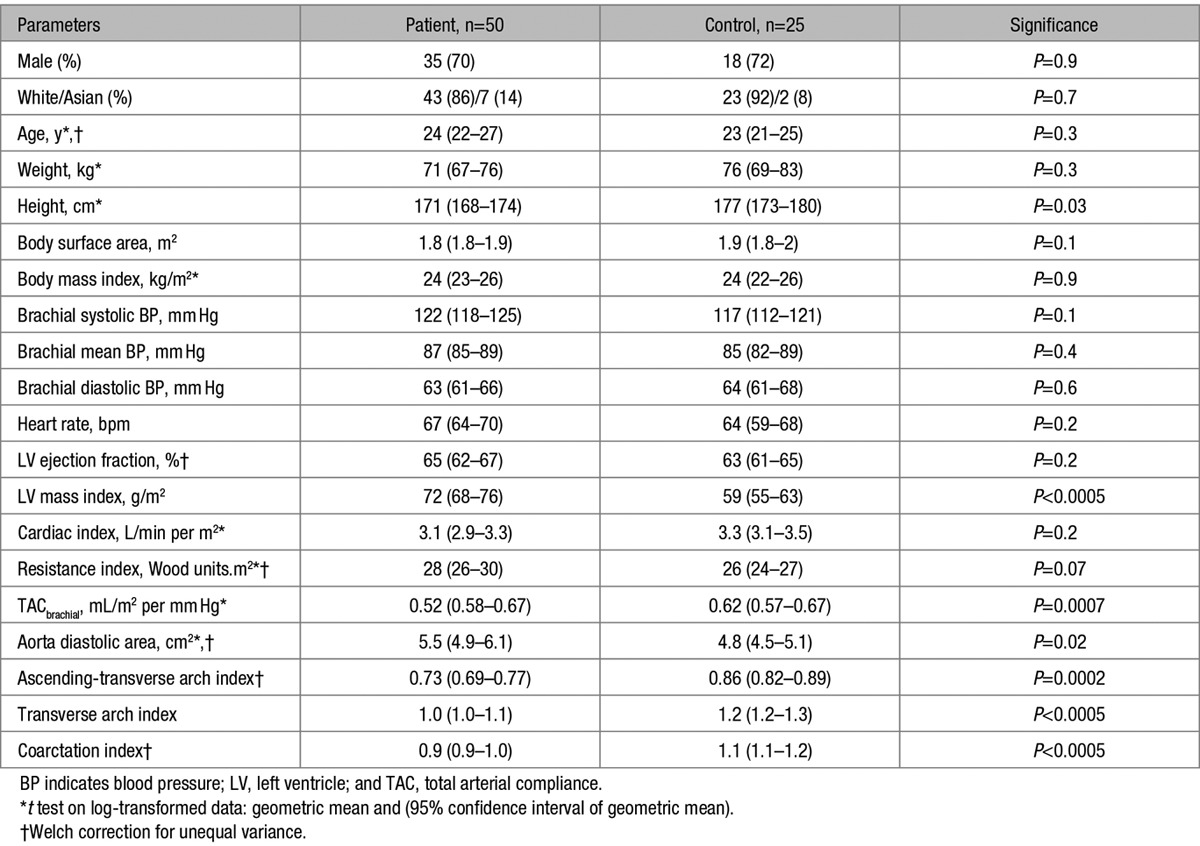
Study Population Demographics and Basic Hemodynamics

The clinical characteristics of the coarctation patients are shown in Table S1. The median age at repair was 4.0 months (interquartile range 0.5–48.2 months). Eighteen of fifty patients (36%) underwent repair in the neonatal period and 34 of 50 (68%) patients had their repair <1 year of age. End-to-end anastomosis was performed in 34 of 50 (68%) patients, extended end-to-end anastomosis in 4 of 50 (8%) patients, subclavian flap angioplasty in 10 (20%) patients, and Dacron/Gore-Tex patch augmentation in 2 (4%) patients. A bicuspid aortic valve was present in 26 of 50 (52%) patients. Eighteen percent of patients underwent a second procedure for recoarctation; 20% of patients were receiving stable antihypertensive therapy. A single agent was prescribed in 8 patients and a combination of agents in 2 patients (2 and 4 agents, respectively; Table S1).

There were no cases of recoarctation on CMR, with all cases having a CI >0.7: group median CI was 0.91 (10th–90th^th^ percentile, 0.78–1.14). In keeping with this finding, no patient had diastolic continuation on Doppler assessment in the descending aorta and the mean arch velocity was 2.0 m/s (1.9–2.2 m/s). There were no cases of significant aortic arch hypoplasia with all patients having TI >0.7; the median TI was 1.04 (10th–90th percentile, 0.88–1.23). Ten of fifty patients (20%) had a “gothic” arch. As expected, TI and CI were all significantly lower in patients than controls, Table 1.

### Blood Pressure: Normal Versus Repaired Coarctation Patients

There was no significant group difference (*P*≥0.1) in brachial SBP, mean BP, or DBP (Table 1), and brachial systolic hypertension (p-SBP>140 mm Hg) was only present in 4 of 50 (8%) patients. However, central SBP was significantly higher (*P*=0.01) in patients (113 mm Hg [110–117 mm Hg]) compared with controls (107 mm Hg [103–110 mm Hg]; Table 2) and central systolic hypertension (c-SBP>125 mm Hg)^21^ was present in 8 of 50 (16%) patients. No control subjects had c-SBP>125 mm Hg or p-SBP>140 mm Hg. There was a moderate correlation between p-SBP and c-SBP (*r*^2^=0.64, *P*<0.0001).

**Table 2.**
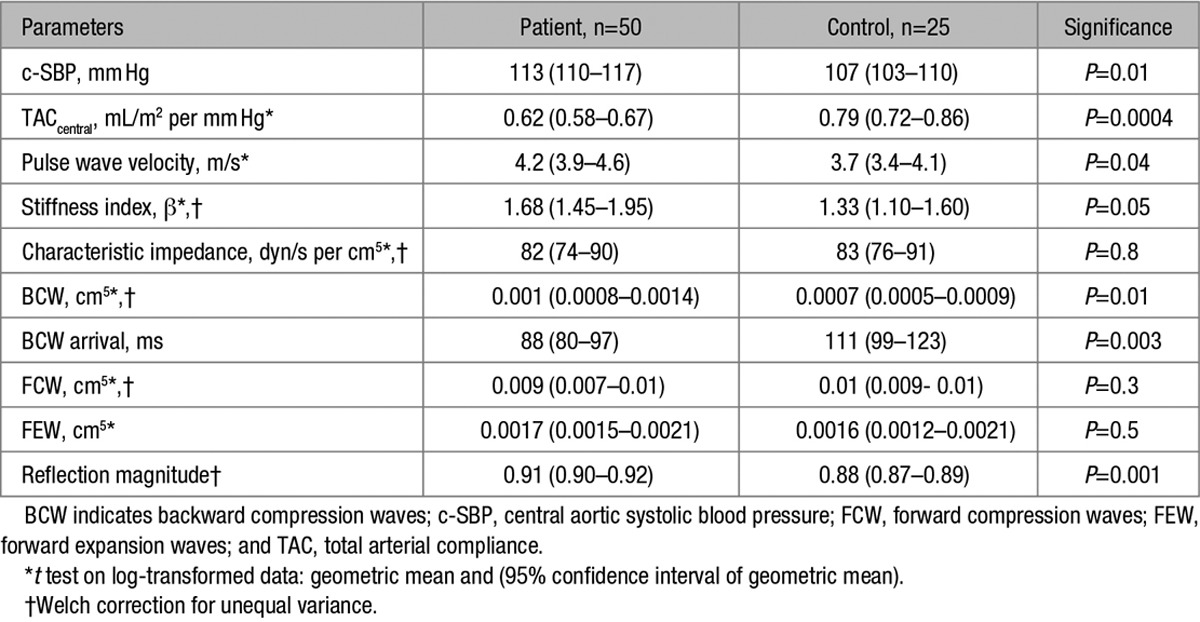
Central Hemodynamics and Wave Intensity

### Conventional Vascular Measures: Normal Versus Repaired Coarctation Patients

TAC calculated using both brachial and central pressures was lower in patients compared with normal controls. However, differences in compliance were more significant when calculated using central, rather than brachial pulse pressure. In addition to evidence of global stiffening, there were also signs of increased ascending aortic stiffness. Specifically, local pulse wave velocity was greater in patients compared with controls (Table 2). However, although patients had a stiffer aorta, the root size was greater, such that no differences in overall aortic root *Z*_c_ were present and, therefore, the pulsatile load imposed by the aortic root was similar in the groups. There was no significant difference in cardiac index or systemic vascular resistance index between the groups, Table 1.

### WIA: Normal Versus Repaired Coarctation Patients

**Figure 1.**
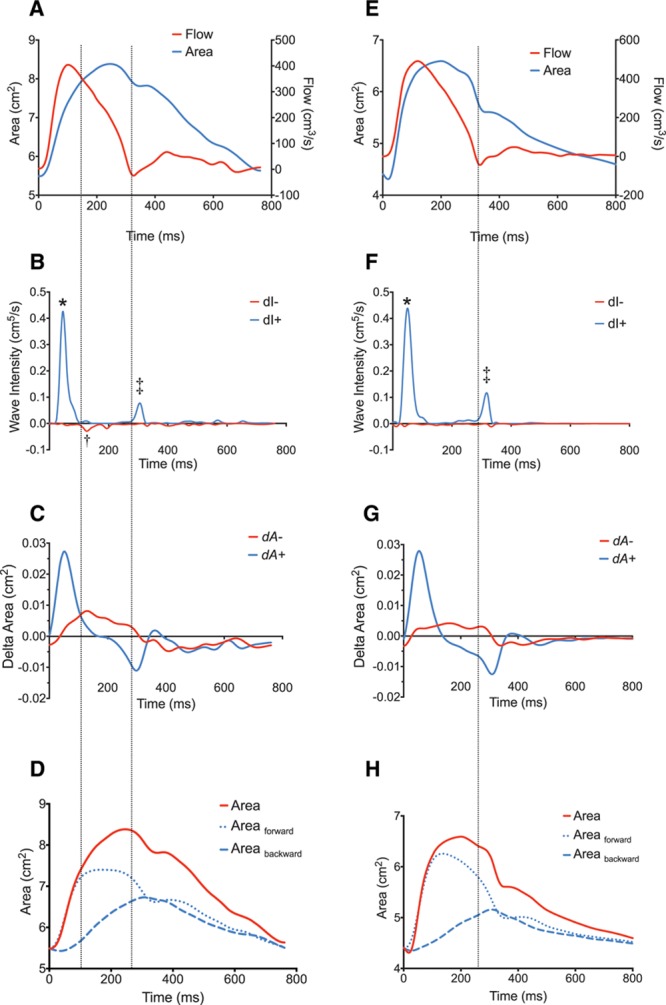
Wave intensity analysis in representative repaired coarctation patient (**A**–**D**) and Control (**E**–**H**). Three main types of waveforms were found to arise during systole in study participants using wave separation analysis: (1) A forward compression wave, characterized by: increasing area and increasing flow representing cardiac ejection, **B** and **F**, labeled “*” (2) A protodiastolic forward expansion wave: decreasing area (pressure) and decreasing flow, **B** and **F** labeled “‡,” and (3) A backwards compression wave: increasing area (pressure) and decreasing flow, **B** labeled “†” (not seen in **F** in this particular control). The identification of the waves as compression or expansion can be seen from examination of **C** and **G**, showing the dA±plots. Time=0 corresponds to the onset of data acquisition as triggered by the R wave on cardiovascular magnetic resonance vectorcardiograph. **D** and **H**, Conventional wave separation analysis with the area waveform separated into forward and backward area waveforms. Vertical dotted lines added to assist visualization of wave timing.

**Figure 2.**
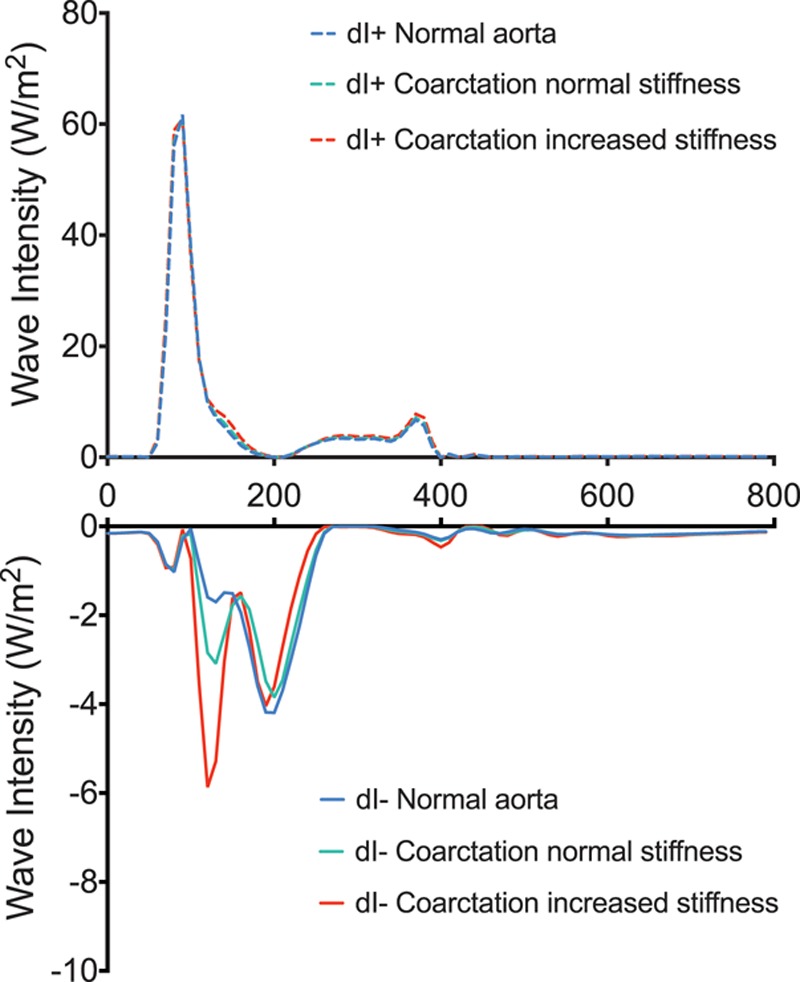
Positive (dI+) and negative wave intensity (dI−) in 1-dimensional simulation of (1) normal aorta (blue), (2) repaired coarctation, coarctation index [CI], 0.75 with normal isthmus stiffness (green), and (3) repaired coarctation, CI, 0.75 with stiff aortic isthmus (red). Units of wave intensity are conventional W/m^2^. Note scale of dI− increased relative to dI+ to assist visualization.

WIA revealed the presence of a midsystolic, BCW, Figure 1. The magnitude of the BCW was significantly higher in patients (0.001 cm^5^ [0.0008–0.0014]) compared with controls (0.0007 cm^5^ [0.0005–0.0009]), (*P*=0.01). In addition, the BCW arrived significantly earlier (*P*=0.003) in patients (88 ms [80–97]) compared with controls (111 ms [99–123]). The magnitude of the BCW was higher in subjects categorized as centrally hypertensive (c-SBP>125 mm Hg), 0.0017 cm^5^ versus 0.0008 cm,^5^ *P*=0.02. There was a significant linear relationship between BCW_log_ and c-SBP (β=0.24, *P*=0.005) after adjustment for body size, age, sex, and TAC_central_. There were no significant differences in the magnitude of the forward compression wave or forward expansion wave between patients and controls (Table 2). These data are in agreement with the results of the 1D model. Specifically, the observed data are similar to the early additional BCW that was observed in the presence of a mild coarctation (25% stenosis). However, the amplitude was significantly higher when the area of the coarctation was stiffer than normal aortic tissue, simulating fibrous scar (Figure 2).

### Arch Morphology

In patients, there was no significant relationship between either CI or arch index and c-SBP (*P*=0.07 and 0.17, respectively), TAC_central_ (*P*=0.29 and 0.95) or BCW (*P*=0.14 and 0.06). There was no significant difference in these metrics in subjects with a gothic arch.

### Determinants of LVM

There was a significant difference (*P*<0.00005) in LVM index between patients and controls, 72 g/m^2^ (68–76 g/m^2^) versus 59 g/m^2^ (55–63 g/m^2^). There was no difference in LVM index between patients with bicuspid and those with tricuspid aortic valves (*P*=0.7).

The determinants of LVM were assessed using univariable linear regression analysis. Models were adjusted for known predictors of LVM: BSA, age, and sex, associations shown in Table 3.

**Table 3.**
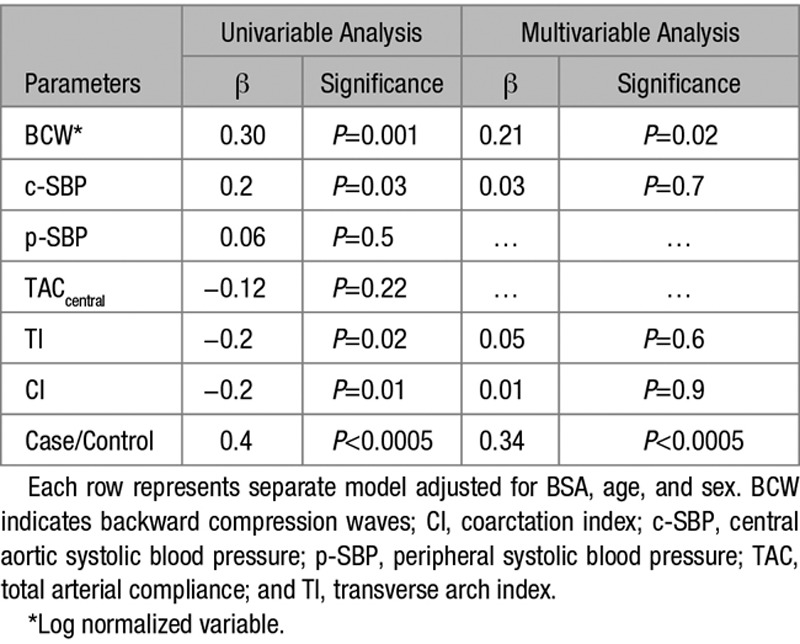
Univariable and Multivariable Relationships of Covariates With LV Mass (g)

On multivariable regression analysis, including significant measures from univariable analysis, only BCW_log_ had a significant independent association with LVM (*P*=0.013; (Table 3). There was no significant independent association of LVM with c-SBP, TI, or CI.

## Discussion

In this study, we used advanced CMR techniques to simultaneously assess all the components of afterload (vascular resistance, TAC, pulse wave velocity, and wave reflections) in patients with repaired coarctation and no significant recoarctation. The main findings of the study were (1) central SBP was higher in patients compared with controls, although there was no significant difference in brachial SBP; (2) patients postcoarctation repair were characterized by lower TAC, higher local wave speed, and increased BCW, without differences in aortic root *Z*_c_; and (3) higher LVM was observed in patients and was significantly associated with the magnitude of the BCW.

### Arterial Stiffness

The simplest measure of afterload is SBP. However, assessment of SBP alone does not specify which components of afterload are abnormal. Therefore, we assessed total and local arterial stiffness, as well as resistance and cardiac output in patients with repaired coarctation. The main findings were reduced TAC and increased ascending aortic stiffness consistent with a postrepair aortopathy. Abnormal aortic stiffness after repair^10,22^ is a consistent finding across studies, and it is confirmed in this study. One obvious cause of reduced TAC is the coarctation repair site itself, which is essentially stiff fibrous scar. However, ascending aortic stiffness (measured remotely from repair site) was also elevated, suggesting a more generalized remodeling phenomenon. Remodeling may also occur as a compensatory mechanism to restore tensile stress to homeostatic levels. Interestingly, local characteristic impedance was not increased in patients because of the opposing effects of wall stiffening and aortic root dilation. This emphasizes that the observed differences in pulsatile load are more related to abnormalities in the distal portions of the aorta (such as the repair site). A limitation of this study was that local vessel properties distal to the repair site were not assessed.^23^ Consequently, it is not known whether descending aortic stiffness is also increased and this is an important area to investigate in a future study.

### Wave Intensity Analysis

The presence of abnormal wave reflection has not previously been assessed in patients with repaired coarctation. Therefore, the increased magnitude of the BCW in patients is an important and novel finding. In patients with significant recoarctation, anatomic impedance mismatching at the site of stenosis would result in wave reflection. In this study, patients with recoarctation were specifically excluded and there was no significant relationship between BCW magnitude and anatomic narrowing. Therefore, an alternative mechanism for the generation of BCWs is required. To investigate possible causes, we used a 1D computer model of the vasculature that can replicate basic pressure and flow physiology. In a model with a mild anatomic narrowing, a small additional BCW was produced. However, only after increasing the stiffness of the repair site did the magnitude of BCW reflect the in vivo findings. Stiffening of the repair site is consistent with fibrous scar,^24^ and our findings suggest that this is an important mechanism in the generation of wave reflections.

It should be noted that there were some important differences between the in vivo and 1D model results. For instance, in the modeled WIA data, there is an early backwards expansion wave that is not present in the in vivo data. However, as this phenomenon has not been demonstrated in any previous in vivo studies, we believe it is an artifact of the model itself. A more important difference is related to the morphology of the BCW. In the modeled data, patients seem to have 2 backwards compressions waves. The first is presumably related to reflections from the coarctation repair site, whereas the second later wave (also present in modeled controls) is likely the result of more peripheral vasculature reflections. In contrast, most patients have only one BCW, although it did arrive slightly earlier than in controls. One possible explanation for this disparity is the relatively low temporal resolution of phase-contrast magnetic resonance, which may cause summation of the 2 backwards waves present in most patients. This would result in the appearance of an earlier arriving, higher magnitude single BCW in patients compared with controls. Wave entrapment and vascular horizon effects could further increase wave summation. This would prevent resolution of separate waveforms, but could explain the earlier arrival of the BCW in patients. In normal subjects, wave entrapment reduces the importance of more distally generated reflected waves. This results in a composite BCW that seems to arise more proximally in the vasculature—the vascular horizon. Because an earlier arriving wave would be more susceptible to summation, wave entrapment might help explain the usual single BCW seen in patients. An earlier arriving BCW could also explain the surprisingly small difference in BCW arrival times in patients versus controls. This could be investigated by performing noninvasive WIA distal to the repair site and would represent an important improvement for future studies. Nevertheless, our findings are consistent with previously published fluid structure interaction models of coarctation. These studies emphasize the role of stiffening as an important possible source of increased BCWs from the arch.^25,26^

In this study, there were no significant differences in the forward compression or expansion waves between patients and controls. However, these waves can modify the morphology of the aortic pressures curve. For instance, the “protodiastolic” forward expansion wave is thought to cause the late systolic inflections that occur in the absence of reflected waves. This was evident in our data (Figure 1), but was not different between the groups. Nevertheless, the magnitude of the forward expansion waves is thought to change with worsening diastolic function and in these patients may be of importance.

### Blood Pressure Differences

We found no difference in brachial measured SBP between patients and controls. Although this is in keeping with previous studies, it does not mean that afterload is normal post coarctation repair. It is well recognized that brachial SBP overestimates central SBP,^21,27^ as seen in our data. This phenomenon is because of patient-specific pressure augmentation and explains the elevated central, but not brachial SBP in our patients. Importantly, a significant proportion of patients would be reclassified as hypertensive using c-SBP. These data suggest that reliance on brachial blood pressure may potentially result in undertreatment of at-risk patients. Failure to address blood pressure adequately in this young population has implications for cardiovascular risk reduction; furthermore, it may also influence cognitive function in later life.^28,29^

Our study is not the first to measure c-SBP after repair of coarctation. Swan et al^30^ measured central blood pressure in postcoarctation repair patients and normal volunteers, and in contrast to our study observed no difference in c-SBP. This may be partly because of their exclusion of any patient with high brachial artery blood pressure. However, another possible reason was their use of the SphygmoCor device to measure c-SBP. This device uses a generalized transfer function to derive central blood pressure from radial tonometric data. The algorithms used are based on a noncongenital population and may not be valid in subjects with abnormal arterial function. In particular, generalized transfer functions may not adequately control for the abnormal wave reflections that may be present in patients post coarctation repair.^31,32^ We used a novel CMR technique to derive c-SBP that is based on exponential modeling of the aortic distension curve.^1^ As this method uses patient-specific data as its initial starting point and for calibration, it should be able to better model pressure amplification.

### Arch Morphology

We found no difference in any hemodynamic parameters between patients with and without “gothic” aortic archs.^10^ Our data were acquired at rest, and studies have observed greater exercise hypertension in patients with a “gothic arch.”^33^ However, Ntsinjana et al^34^ using quantitative (rather than qualitative) analysis of arch angulation found no association with exercise blood pressure after adjustment for CI.

### Left Ventricular Mass

In this study, patients with repaired coarctation had elevated LVM, which has been shown to strongly predict increased cardiovascular morbidity and mortality.^35,36^ In multivariable analysis, the most important hemodynamic association with LVM was the magnitude of the BCW. This is in keeping with previous human and animal studies that have shown that wave reflections play a prominent role in determining LV structure and function. In the multiethnic study of atherosclerosis, backward waves have been shown to be associated with both LVM^37^ and heart failure events.^38^ Furthermore, Hashimoto et al^39^ identified that antihypertensive therapy reduced backward waves, which may have an important role in mediating the ventricular response to therapy. The exact mechanism by which early BCWs contribute to the development of increased LVM is uncertain. However, it may be related to reflected waves altering the temporal evolution of LV pressure. For instance, Kobayashi et al^40^ showed a more profound hypertrophic response in rats banded in the descending aorta compared with the ascending aorta. This was thought to be because of late systolic loading due to wave reflections being a more potent hypertrophic stimulus. Irrespective of the mechanism, these data demonstrate that afterload cannot simply be thought of as SBP.

### Novel CMR Imaging

Comprehensive noninvasive assessment of hemodynamics was made possible in this study because of the novel CMR and signal processing techniques used. All of the hemodynamic data were derived from a single high temporal-resolution phase-contrast acquisition, obtained during a breath-hold. These data provide the prerequisite area and flow data needed to derive central systolic pressure, resistance, TAC, local pulse wave velocity, and wave intensity when combined with simultaneous brachial noninvasive blood pressure measurement.

In this study, TAC was assessed using a technique based on 2-element Windkessel modeling. This technique is known to be an improvement on the stroke volume/pulse pressure method, which overestimates TAC. However, this technique has been criticized because it does not account for wave reflections. Nevertheless, it does provide and easily understood metric of arterial buffering that captures the simpler elements of pulsatile load. Consequently, we believe that it is a useful adjunct to the more sophisticated WIA performed in this study.

We also used the Bramwell–Hill equation for the derivation of local pulse wave velocity/characteristic impedance, rather than the flow-area (QA) or impedance-based methods. This was because we anticipated significant early reflections during the early part of systole, which would have resulted in errors in estimation of pulse wave velocity.^15^

WIA provides useful insight into wave reflections in the time domain, it is relatively simple to implement but requires high temporal-resolution data flow imaging to resolve waveforms, which have short durations.

The ability to assess c-SBP, TAC_central_, and characteristic impedance during routine CMR assessment is feasible, whereas performing noninvasive WIA may be more challenging to implement. However, the association with LVM suggests that these parameters may be useful biomarkers. Further studies assessing their relationship to outcomes and response to therapies are necessary.

### Limitations

A significant limitation of this study is the possibility of cascading errors resulting from inaccuracies in the multiple acquisition and processing steps required to perform noninvasive WIA. These errors could originate from residual aliasing in the reconstructed images, incorrect segmentation, inaccurate blood pressure measurement, and inadequate assumptions. Nevertheless, the relationship between LVM and BCW does add credence to our findings.

Central SBP was derived by calibrating aortic area curves to mean and DBP using an exponential pressure–area relationship. This method was originally validated using noninvasive carotid tonometry (over a similar pressure range in this study), rather than using preferred invasive pressures. Unfortunately, micromanometer pressure catheters are not available for the magnetic resonance imaging environment and the alternative, fluid-filled catheters, have unfavorable frequency response and damping characteristics, which limit their use for this purpose. We have also previously described the use of a more complicated “arctangent” pressure–area model; however, this method cannot be reliably calibrated using noninvasive local pulse wave velocity (Q/A method), as it underestimates wave speed in the presence of significant early wave reflections, as observed in coarctation patients.^15^

The units of noninvasive WIA in this study, cm^5^ do not have an easily understandable physical meaning: in contrast to the W/m^2^ units of conventional WIA. Nevertheless, the waveforms produced by noninvasive WIA are qualitatively similar to invasive WIA in the literature—and given a linear pressure–area relationship would be directly proportional. Area and flow waves can, therefore, be considered analogous to pressure and velocity waves as found in the WIA literature.

## Conclusions

This study aimed to describe all relevant central hemodynamic parameters underlying the vascular abnormalities in CoA. We have shown that this can be performed within a single breath-hold, using a high temporal-resolution phase-contrast acquisition in the ascending aorta with simultaneous oscillometric blood pressure measurement. These data provide central blood pressure, systemic vascular resistance, TAC, local pulse wave velocity, and wave reflections.

We have shown that patients with repaired coarctation have higher c-SBP, stiffer aortas, and abnormal wave reflections, despite similar p-SBP to controls. Elevated LVM in this population is associated with the magnitude of the BCW. Consequently, therapies that can influence TAC and wave reflections may represent important drug development targets for the future.

## Perspectives

Understanding the vascular abnormalities in patients with CoA is important if we wish to modify the cardiovascular risk and excess mortality in this population.^41^ Conventional clinical assessment using brachial blood pressure and anatomic imaging may fail to identify patients with vascular dysfunction, thereby leading to inappropriate undertreatment. Future therapeutic interventions should be targeted to the vascular abnormalities, which occur in this population.

## Sources of Funding

This research has been supported by the British Heart Foundation (MQ [FS/11/76/29037] and [FS/16/28/32327]) and the National Institute for Health Research, Great Ormond Street Hospital, UCL Biomedical Research Centre award.

## Disclosures

None.

## Supplementary Material

## Novelty and Significance

### What Is New?

- Patients with repaired coarctation of the aorta have an aortopathy associated with elevated central systolic blood pressure, decreased total arterial compliance, increased local pulse wave velocity, and increased backwards compression waves, without differences in aortic root characteristic impedance.
- The magnitude of the backward compression wave is independently associated with LV mass.
- Indices of conduit vessel function can be measured noninvasively using a combination of phase-contrast magnetic resonance imaging and oscillometric sphygmomanometry. This assessment could be appended to routine magnetic resonance imaging surveillance typically used in this population.

### What Is Relevant?

- Currently used indices, such as p-SBP, coarctation index, and arch index, may not adequately describe vascular dysfunction in this population.

### Summary

Comprehensive evaluation of vascular resistance, total arterial compliance, local pulse wave velocity, and wave reflections can be performed noninvasively. Measurement of these parameters helps define the vascular abnormalities in patients with repaired coarctation of the aorta. Therapeutic targeting these abnormalities may more effectively reduce cardiovascular risk in this population.
