# Supplementary material for: Abnormal Wave Reflections and Left Ventricular Hypertrophy Late After Coarctation of the Aorta Repair
Source: Hypertension. 2017 Feb 8;69(3):501–9. doi: 10.1161/HYPERTENSIONAHA.116.08763 (PMC5295491; doi:10.1161/HYPERTENSIONAHA.116.08763)
Supplement: Supplementary file 1 [file hyp-69-501-s001.docx]

**Supplement**

**Abnormal Wave Reflections and LV Hypertrophy Late After Coarctation of the Aorta Repair**

Michael A. Quail, MB ChB, PhD^1^; Rebekah Short, BSc^1^; Bejal Pandya, MBBS^1,2^; Jennifer A. Steeden, PhD^1^; Abbas Khushnood MBBS^1^; Andrew M Taylor MD^1^, Patrick Segers, PhD^3^; Vivek Muthurangu, MD^1^.

1. Centre for Cardiovascular Imaging, Institute of Cardiovascular Science, University College London and Great Ormond Street Hospital for Children, WC1N 3JH, UK.
2. Adult Congenital Heart Disease Department, St Bartholomew’s Hospital, London, EC1A 7BE, UK.
3. IBiTech-bioMMeda, iMinds Medical IT, Ghent University, De Pintelaan 185, 9000 Gent, Belgium.

*Supplemental Methods*

**CMR Protocol**

*LV Volume and Mass*

Images were acquired in the ventricular long-axis, four chamber, and the short axis covering both ventricles (9-12 slices). Assessment of the LV volume was performed by manual segmentation of the endocardial contour of short-axis cine images at end-diastole and end-systole using a built-in plug-in for OsiriX. End diastolic volume and end systolic volume were calculated using Simpson’s rule. From these volumes, stroke volume and ejection fraction (EF) were calculated. Epicardial contours were manually segmented at end systole. Ventricular mass was calculated as the difference between the epicardial and endocardial contours multiplied by the slice thickness and a specific gravity of ventricular mass of 1.05g/ml. LV mass was adjusted for body surface area (BSA) to provide the indexed LV mass (g/m^2^).

*Aortic Arch Anatomy*

Aortic arch morphology was assessed in patients using gadolinium-enhanced MR angiography as previously described with a coronal 3D fast-field-echo sequence.^1^ Gadolinium (Dotarem, Guerbet) was injected into a peripheral vein and tracked into the heart with a dynamic coronal 2D fast-field-echo sequence. The gadolinium dose was 0.2 mmol/kg. The MR angiographic sequence was started when contrast reached the left ventricle. Two consecutive angiograms were acquired in a single 15- to 20-second period of apnea. In control subjects and 7 patients intravenous contrast was not administered (IV access could not be obtained [n=2] or no consent for contrast [n=5]). Aortic anatomy was assessed in these subjects using a balanced, steady-state free precession sequence. ^2^

*Image Processing*

All images were processed using an in-house plug-in for the open source DICOM software OsiriX (OsiriX Foundation, Geneva, Switzerland).^3^ Segmentation of the ascending aorta was performed on the modulus image using a previously validated semi-automatic registration-based algorithm.^4^ The aortic region of interest (ROI) was manually adjusted as necessary to ensure optimal vessel wall delineation. The final ROIs were used to both calculate the aortic cross-sectional area (A) and prescribe the region in the phase image from which mean velocity (V) and flow (Q) were calculated. However, pressure wave synthesis requires curves that span the whole RR interval and the use of prospective gating resulted in missing data in the last 80-100ms of diastole. These missing data were recovered using linear interpolation between the last point and the first point. The curves were then linearly interpolated to 1ms temporal resolution and filtered using a zero-phase, low-pass, 2^nd^ order Butterworth filter with normalized cut-off frequency of 0.04 (20Hz), (Matlab 2014b, Mathworks).

Aortic arch anatomy was evaluated by measurement of aortic diameter in the transverse aortic arch between innominate and left common carotid artery, distal aortic arch (repair site), and the descending aorta at the level of the diaphragm. Two metrics were derived from these measurements (Figure S1): (1) Transverse arch index (TI), quantifying the degree of transverse arch hypoplasia: transverse arch diameter divided by descending aorta diameter. (2) Coarctation index (CI), quantifying the degree of recoarctation: aortic isthmus (repair site) diameter divided by descending aorta diameter. Aortic arches were also characterised as ‘gothic’ if the arch had an acutely angulated triangular conformation.^5^

*Wave Intensity Analysis*

In WIA, waves are regarded as a summation of incremental wave fronts; it is therefore possible to separate the *Q* and *A* curves into the respective forward (+) and backward (-) components by expressing the relationship between wave speed, *c* (Equation 1) and changes in flow and cross sectional area.

$c = \pm\frac{{dQ}_{\pm}}{{dA}_{\pm}}$ Equation 1

Equation 1 combined with Equations 2 and 3:

$dA={dA}_{+}+ {dA}_{-}$ Equation 2

$dQ={dQ}_{+}+ {dQ}_{-}$ Equation 3

can be solved for the changes in the forward and backward flow and cross-sectional area; this results in Equations 4 and 5:

${dQ}_{\pm}=\frac{1}{2}\left( dQ\pm cdA \right)$ Equation 4

${dA}_{\pm}=\frac{1}{2}\left( A\pm\frac{1}{c}dQ \right)$ Equation 5

Net wave intensity *dI*_a_ was defined as the product of the differentials of cross-sectional area and flow.

${dI}_{a}=dA dQ$ Equation 6

Similarly it can be shown that the net wave intensity *dI*_a_ (Equation 6) can be divided into the forward and backward intensities, Equation 7:

${dI}_{a}={dI}_{a (+)}+ {dI}_{a (-)}$ Equation 7

with the separated *dI*_a_ expressed as:

${dI}_{a (\pm)}= \pm\frac{c}{4}\left[ dA\pm\frac{dQ}{c} \right]^{2}$ Equation 8

Using this formulation and the value of c calculated using the Bramwell-Hill equation, forwards and backwards *dI_a_* were calculated and plotted. As per convention, the direction of waves was referenced to the direction of blood flow. Waves arising from the heart were defined as forward running and those arising from the vasculature as backward running. Waves causing an increase in area were classified as compression waves and those causing a decrease in area as expansion waves by examination of dA_±_ plots. Thus, a forward running wave was held to be a compression wave if dA_+_ was greater than zero and an expansion wave if dA_+_ was less than zero. Similarly, a backward-running wave was considered as a compression wave if dA_−_ was greater than zero and an expansion wave if dA_−_ was less than zero.

Using this system four different waves may be characterised: Forwards Compression Waves (FCW), Forwards Expansion Waves (FEW), Backwards Compression Waves (BCW) and Backwards Expansion Waves (BEW).

The type of wave and their magnitude (area under the wave) were determined by analysis of the net and separated WIA plots in Matlab. The areas under the separated waveforms were calculated by numerical integration. Area waveforms were also separated into forward and backward components, by integration of dA_+_ and dA_-_ plots. Using these data, we calculated the reflection magnitude as: Area_backward_/Area_forward_.

Supplement References

1. Muthurangu V, Taylor AM, Hegde SR, Johnson R, Tulloh R, Simpson JM, Qureshi S, Rosenthal E, Baker E, Anderson D, Razavi R. Cardiac magnetic resonance imaging after stage i norwood operation for hypoplastic left heart syndrome. *Circulation*. 2005;112:3256-3263.

2. Quail MA, Nordmeyer J, Schievano S, Reinthaler M, Mullen MJ, Taylor AM. Use of cardiovascular magnetic resonance imaging for tavr assessment in patients with bioprosthetic aortic valves: Comparison with computed tomography. *Eur J Radiol*. 2012;81:3912-3917.

3. Rosset A, Spadola L, Ratib O. Osirix: An open-source software for navigating in multidimensional dicom images. *J Digit Imaging*. 2004;17:205-216.

4. Odille F, Steeden JA, Muthurangu V, Atkinson D. Automatic segmentation propagation of the aorta in real-time phase contrast mri using nonrigid registration. *J Magn Reson Imaging*. 2011;33:232-238.

5. Ou P, Celermajer DS, Raisky O, Jolivet O, Buyens F, Herment A, Sidi D, Bonnet D, Mousseaux E. Angular (gothic) aortic arch leads to enhanced systolic wave reflection, central aortic stiffness, and increased left ventricular mass late after aortic coarctation repair: Evaluation with magnetic resonance flow mapping. *J Thorac Cardiovasc Surg*. 2008;135:62-68.

| Age of Repair  Neonatal Repair  Repair <1year | 18 (36%)  34 (68%) |
| --- | --- |
| Type of Repair  End-to-End Anastomosis  Extended End-to-End Anastomosis  Subclavian Flap Angioplasty  Dacron/Gore-Tex Angioplasty | 34 (68%)  4 (8%)  10 (20%)  2 (6%) |
| Secondary Re-intervention  Balloon Angioplasty  Surgical Angioplasty | 7 (14%)  2 (4%) |
| Antihypertensive Medication  ACE inhibitors  Angiotensin Receptor Blockers  Calcium Channel Blockers  Beta Blockers  Thiazide Diuretics | 10 (20%)  4 (8%)  4 (8%)  3 (6%)  2 (4%)  1 (2%) |
| Additional Cardiac Lesions  Bicuspid Aortic Valve  Ventricular Septal Defect  (resolved/repaired) | 26 (52%)  19 (38% |
| Additional Clinical Abnormality  Turner’s Syndrome/Mosaic XO | 2 (4%) |

Table S1 Coarctation patient clinical demographic information

Figure S1 Location of Aorta measurements. (A) Level of CMR flow measurement: Ascending Aorta above sinotubular junction. (B) Transverse aortic arch between innominate and left common carotid artery. (C) Narrowest distal aortic arch (repair/recoarctation site). (D) Abdominal aorta at diaphragm. Two metrics were derived: (1) Transverse arch index (TI) = B/D and (2) Coarctation index (CI) =C/D.
